# The Temperature of the External Auditory Canal in Disease

**Published:** 1884-12

**Authors:** 


### The Temperature of the External Auditory Canal in Disease.

A notable contribution from the pen of A. Eitelberg, of Vienna, upon the above subject, is found in the current volume of the *Archives of Otology*, based upon measurements of temperature of the external ear in over fifty cases of aural disease. His measurements were made with a delicate thermometer especially constructed and adapted to the purpose. He found "as a rule the temperature of the external auditory canal varied between 36.8° and 37.4° (Centigrade), these limits being exceeded to a marked degree in a few cases only. Increased temperatures in the external auditory canal were not found in any case without a similarly increased temperature of the body." In only one case was the temperature of the ear higher than that of the axilla. This was one of otitis media on the right side. "In tympanic affections of a less active character, the temperature was the same, or nearly the same, as a rule, in the two ears, and the relation between the temperatures of the ear and axilla was normal." When the temperature was considerable higher on the affected than on the healthy side, it did not exceed that of the body at all, or only a trifle, while that of the normal ear was lower than that of the body. Febrile temperature of the body is also accompanied with febrile temperature in the external auditory canal.

The question again arises, whether the temperature at the point of an inflammation is really higher than that of the body.

The facts of the author seem to indicate that it is not. Billroth, in 1865, gave the results of measurements of the temperature of forty-eight wounds. In only two cases was it higher at the point of inflammation than in the rectum. Jacobson and Bernhardt, in 1869, stated that they found, without a single exception, that the temperature of the inflamed pleura and the inflamed peritoneum was lower by several tenths of a degree than that of the heart. They believed that the idea prevalent in modern pathology that inflamed tissue is higher in temperature than the body was erroneous, and that John Hunter's dictum, that local inflammation does not increase the temperature of any part beyond the temperature of the organ of circulation, was correct.

